# Does perceived organizational support matter? The effect of high-commitment performance management on supervisors’ performance

**DOI:** 10.3389/fpsyg.2022.837481

**Published:** 2023-01-11

**Authors:** Mohammad Rabiul Basher Rubel, Daisy Mui Hung Kee, Yahya Qasim Daghriri, Nadia Newaz Rimi

**Affiliations:** ^1^Department of Business Administration in Management Studies, Bangladesh University of Professionals, Dhaka, Bangladesh; ^2^School of Management, University of Science Malaysia, Penang, Malaysia; ^3^Department of Management, Faculty of Business Studies, University of Dhaka, Dhaka, Bangladesh

**Keywords:** high-commitment performance management, high-commitment HRM, job performance, in-role behavior, extra-role behavior, deviant behavior

## Abstract

The current research aims to investigate the connection between supervisors’ perceived high-commitment performance management (HCPM) and their performances (in-role, extra-role, and deviant work behavior). In addition, this paper aims to examine how perceived organizational support (POS) mediates the above relationship. The paper employs the social exchange theory as the theoretical lens to develop and suggest a positive motivational work environmental model. Our model is tested on a sample of 430 supervisors from ready-made garment (RMG) organizations, Bangladesh. Using the PLS-SEM, our model examines the direct and indirect effects of HCPM and POS on job performances. We find supports for the existence of a positive relationship from HCPM to job performance through POS mediating such a relationship. Future studies may investigate the prospective of HCPM and POS to create ideal work environments that boost employee productivity and benefit enterprises.

## Introduction

High-commitment performance management (HCPM) has positive benefits for both employees and the organization (Latorre et al., 2016, 2020; Rubel et al., 2021). HCPM comprises performance management approaches such as taking employee input for performance target setting, assessing performance and providing appraisal feedback, choosing pay and benefit alternatives for better performance, and creating development opportunities and new targets that are aimed to foster employee commitment through their involvement and personal development (Farndale et al., 2011). Den Hartog et al. (2004) argued that HCPM could affect employees’ perceptions of organizational actions, impacting employee attitudes and behaviors. We view HCPM as an ongoing, positive motivational tool that enables an organization to manage employee performance taking social exchange theory (SET) into consideration. We expect that HCPM would encourage a reciprocal relationship between employees and employers, promoting positive behaviors and minimizing negative behaviors from the employees. We propose that HCPM may directly lead to positive employee performances such as increased in-role and extra-role behaviors and decreased deviant work behaviors. In addition, we anticipate an indirect influence of perceived organizational support (POS) in the link between HCPM and employee performances, here supervisors are the concerned employees.

This paper focuses on HCPM, a subsystem of practices within HCHRM. Farndale et al. (2011) examine why and how performance management (PM) can be approached as one dimension of HCHRM and termed HCPM. According to Farndale and colleagues, HCPM practices include employee participation in performance target setting, performance measures leading to development opportunities and new targets, regular appraisal feedback, and choosing pay and benefit options that can engender employees’ commitment through their involvement and personal development.

Empirically, our contribution is threefold. First, we take a new perspective on examining HCPM (a specific dimension of HCHRM) considering its dimensions (goal and participation, performance appraisal, performance feedback, and performance reward) in relation to individual employee behavioral outcomes that comprise both productive (in-role and extra-role behavior) and counterproductive behavior (deviant behavior). The finding of a specific or single practice of the HCHRM is rarely specified or has not much been investigated regarding individual employee outcomes (Rubel and Kee, 2015a). Secondly, this paper illustrates HCPM as a higher-order or hierarchical reflective model contributing to this study. Therefore, this study serves as a platform for identifying the hierarchical modeling of individual dimensions of HCHRM practices based on which an organization can identify the possible dimensions under that practice determined through organizational and other contextual differences. Finally, our paper adds to the HCPM literature, proposing a mediating influence of POS on the relation between HCPM and employee performances. The topic is important and deserves further research as today’s organizations are still searching for ways to enhance employee work outcomes through both HRM and POS (Shanock et al., 2019; Abdelmotaleb and Saha, 2020). Here, we introduce POS as an intervening variable between HCPM and employee in-role, extra-role, and deviant behaviors.

Earlier literature also employs POS as the predictor of employee behavioral outcomes such as work performance and turnover intention (Shen et al., 2014), job satisfaction (Wen et al., 2019; Côté et al., 2021), employee innovative work behavior (Nazir et al., 2019), employee creativity (Akgunduz et al., 2018), and employee engagement (Imran et al., 2020). Thus, in earlier research, a gap is found where POS is considered the interpreter of employees’ productive (role-prescribed and extra-role behavior) as well as non-productive (deviant behavior) behaviors. Furthermore, in previous research, POS is also found as a mediator between different variables, for instance, high performance HRM and employee work outcomes (Rubel et al., 2020), transactional leadership and employee creativity (Suifan et al., 2018), presenteeism and job satisfaction (Côté et al., 2021), and job stress and organizational commitment (Saadeh and Suifan, 2020). So far studied, there is a dearth of research regarding POS as a mediator between HCPM, an individual dimension of HCHRM practices, and both positive and negative employee outcomes in the workplace, pointing to another motive of the study.

Moreover, this paper examines how HRM influences employee behaviors that needs further contextual proof (Wahid and Hyams-Ssekasi, 2018; Rubel et al., 2020, 2021). In addition to HRM research in the Western context, HRM in developing countries, particularly Asian countries, needs to be explored further (Sultana and Johari, 2022). Indeed, several prominent authors (Cooke, 2018; Cooke et al., 2020) have convincingly reasoned for more Asia-oriented HRM research. This article echoes an argument for the HCHRM field in Bangladesh, an emerging Asian economy. This study focuses on the ready-made garment (RMG) business, which has emerged as one of the strongest and fastest growing manufacturing sectors in Bangladesh’s economy that is now the world’s third-largest garment exporter, trailing only China and Vietnam (Bangladesh Bureau of Statistics, 2022). However, the RMG industry is facing tremendous challenges in employee performance stability, labor unrest, and high turnover that are the potential threats to the sustainable growth trend of this industry (Ullah et al., 2013; Hasan et al., 2018). Local research has confirmed that HR enabling factors, namely HR practices such as empowering employees, competency development, employee’s acceptance to change culture, team working facilities, and valuable performance appraisal, are the possible solutions to address these HR-oriented challenging issues of the RMG industry (Alam et al., 2018; Talapatra et al., 2019). Being motivated by such findings, we examine if HCPM could improve RMG employee performances by creating positive organizational perceptions.

**Figure 1:**
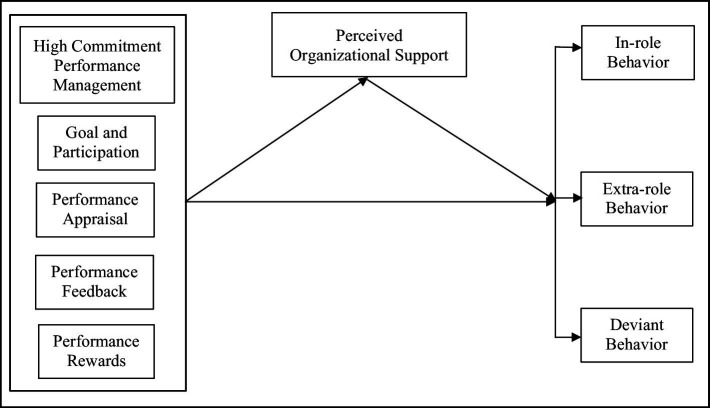
Our research model.

Recognizing the benefits of HRM practices as predictors of workplace productivity (Mostafa et al., 2015; Khoreva and Wechtler, 2018; Veth et al., 2019; Rubel et al., 2020), the need to establish an HR-focused framework that promotes better job performances in the workplace is emerging. This paper integrates an additional element, HCPM, an organizational-based resource that likely explains each job performance dimension (in-role, extra-role, and deviant behavior). We broaden our investigation by looking at the effect of HCPM on Bangladeshi RMG supervisors’ job performance *via* POS. RMG employee performance is critical so that business processes can be further improved, given that the RMG industry is labor-intensive (Hossan et al., 2012; Rubel and Kee, 2015b; Hossain and Mahmood, 2018; Rubel et al., 2020). Within such a context, examining RMG supervisors’ perceptions of HCPM and how such perceptions work for positive performances is essential to comprehend how they could implement HCPM to ensure their subordinates’ positive organizational supports and task performances. We aim to contribute to the current HCHRM literature by examining how the perceptions of HCPM enhance employee job performances. We investigate POS as an intervening variable in the aforementioned interaction and answer how HCPM relates to employee behavioral outcomes in the Bangladeshi RMG industry to provide contextual proof of HRM study. Figure 1 depicts our model.

### Ready-made garment industry in Bangladesh

Bangladesh’s RMG business is widely acknowledged as a major contributor to the country’s economic progress (Hoque et al., 2021). Despite the industry’s tremendous growth, Bangladeshi garment factories struggle to meet the minimum standard imposed by national and international regulatory bodies (Rubel et al., 2017; Maalouf et al., 2019). Because of the unfavorable working conditions, garment workers are dissatisfied, their turnover rate is high, labor unrest is a normal phenomenon, and the industry has been thrown into chaos by workers demanding their due rights (Hasan et al., 2018). Inadequate health and safety programs, indifferences of the owners to respond to workers’ problems, less worker engagement in decision-making, less number of training, and insufficient regulating and monitoring are just a few of the major causes of worker dissatisfaction and conflict (Rubel et al., 2017; Alam et al., 2018; Maalouf et al., 2019; Roy, 2020). Employees in this industry have attacked factories, overturned vehicles, and blocked roads as a show of displeasure with the company’s terrible labor standards (Roy, 2020).

Employee turnover is a concern in the RMG industry due to discontent with work facilities such as pay structure, leave policy, medical coverage, advancement opportunities, and working environment (Rubel et al., 2017; Elahi et al., 2019). Employees at RMG are not assessed based on their contributions, and they do not have access to the essential and entitled benefits, which exacerbates their dissatisfaction and leads to unfavorable attitudes and conduct (Rahman and Chowdhury, 2020). RMG, being a labor-intensive industry, need to resolve these labor issues. To this purpose, local studies suggest that important yet unsolved problems in labor management, particularly in valuing and acknowledging employees’ contributions to the industry, be addressed (Ahmed et al., 2013). The causes of worker unhappiness or discontent may impact employees’ task performance and the garments’ overall performance. RMG firms must promote their employees’ task performances in order to fulfill buyers’ rising demand for a reasonable price, shorter lead time, and high product quality (Hoque and Shahinuzzaman, 2021; Hoque et al., 2021). Previous studies demonstrate that decision-making through employee participation, appropriate performance evaluation and monitoring, and satisfactory rewards and recognition might be the options for garment manufacturers to improve their working environment and individuals’ task performance (Yang and Maresova, 2020; Hoque and Shahinuzzaman, 2021). We propose that HCPM comprising goal and participation, performance appraisal, performance feedback, and performance reward may relate to the increased performance and decreased unproductive performance of RMG employees. Using this logic as a starting point, we investigate how HCPM relates to in-role, extra-role behavior, and deviant behavior through POS.

### Social exchange theory

An employment relationship essentially portrays social exchange characteristics conceptualized in SET (Rubel et al., 2018). SET states that the exchange partner’s benefits are returned in a discretionary way and create a long-term continuing relationship (Blau, 1964). Likewise, the employment relationship begins and develops over time based on benefits exchanges between employees and the organization (Cropanzano and Mitchell, 2005). HRM is undoubtedly one of the most successful organizational strategies for indulging exchange behavior in organizations and SET structures employee-organization connections in a benefit exchange form (Rubel et al., 2018; Mohammad et al., 2021). Superior social exchanges through HRM practices have been linked to more significant employee outcomes (Tuzun and Kalemci, 2018; Katou et al., 2021). HRM, as organizational practices, affects employee responses primarily because it communicates to employees that the organizations are serious about their well-being and appreciate their roles (Neves and Eisenberger, 2012). HCHRM is seen as an input into the social exchange process to shape positive employee attitudes and behaviors (Rubel et al., 2021). Moreover, there is evidence that individual HRM practice impacts employee perceptions of organizational support to solicit employee behaviors in the same way (Snape and Redman, 2010).

SET proposes the subsistence of a two-way behavioral reinforcing relationship. It is believed that HCPM in the forms of employee participation, feedback, and reward symbolizes the organization’s support and social approval of their contribution, which might generate positive performance behaviors and reduce deviant behavior to support the notion of a reciprocal employment relationship. Using SET literature as a theoretical backdrop, we argue that HCPM and POS are available resources to encourage employee in-role and extra-role behaviors within the workplace. Employees who experience high POS are expected to reinvest their efforts back into the organization and are less likely to engage in deviant behavior.

## Literature review and hypotheses

### High-commitment performance management

Performance management is an important component of the HRM process, especially for triggering strong employee commitment (den Hartog et al., 2004; Bellisario and Pavlov, 2018). Farndale et al.’s (2011) study first describes how PM can be approached as HCPM practices. There are two forms of commitment-based PM practices: “involvement centered” and “development centered” (Farndale et al., 2011). Involvement-centered focuses on individual attachment in setting performance goals, discussing and providing performance feedback, and having choices over the rewards (pay and benefits) against the performance. On the contrary, the development-centered emphasizes employees’ appraisal to develop their skills, knowledge, and capacity.

PM is implemented to communicate performance goals, send feedback on actual performance, prompt and reward work behavior, and inspire development action (DeNisi and Murphy, 2017; Pavlov et al., 2017). We conceptualize HCPM into four dimensions: goal and participation, performance appraisal, performance feedback, and performance rewards. Performance goals signal what employees need to do and why; monitoring and feedback provide information about the degree of attainment of their performance goals; performance-based rewards provide “an unambiguous perceived cause-effect relationship in reference to the HRM system’s desired content-focused behaviors and associated employee consequences” (Bowen and Ostroff, 2004: 210). PM should have consistency among these four activities (Audenaert et al., 2019). Such a consistent PM system follows the logic of plan-do-check-act principles of a continuous performance improvement process (Aguinis et al., 2013), authenticated by the interconnected stages of planning, monitoring, feedback, and recognition tasks (Decramer et al., 2013). These PM practices enable employees to realize why and how they are inclined to work for the organization (DeNisi and Smith, 2014).

### Perceived organizational support

Kim et al. (2016) defined POS as employee perceptions on how the organization values their contributions and cares about them. POS encompasses employee perceptions of organizational support toward the performance in one’s job and creates felt obligation to the organization; develops an expectation of reward for increased effort; meets socio-emotional needs, and produces an expectation that help will be available when needed to perform one’s job better (Eisenberger and Stinglhamber, 2011; Rubel et al., 2020). Prior literature suggested that employees with higher POS evaluate their jobs more favorably (e.g., enhanced job satisfaction and empowerment; Maan et al., 2020) and invest more in their organization (e.g., work engagement, employee retention, and organizational commitment; Gupta et al., 2016; Guan and Frenkel, 2018). Latorre et al. (2016) advocated that HCHRM is linked with POS because POS signals a sense of caring and well-being of employees. We propose that each dimension of HCPM (goal and participation, performance appraisal, performance feedback, and performance rewards) would lead to a positive POS. HCPM, an organizational-based resource, would explain how HCPM is vital for workplace resourcing, and this HCPM could have a link to POS.

### Employee behaviors

To achieve organizational goals and objectives, employees’ attitudes and behavior toward a job are critical. However, it is also true that in an organizational setting, employees might behave in different ways, each with different responses toward individuals and the organization (Tuzun and Kalemci, 2018). Employee behaviors are productive when they view the organization as supportive (Appelbaum et al., 2007). In extreme cases, employees might exhibit negative or counterproductive behaviors if they feel that organizations do not support them (Kalemci et al., 2019; Khattak et al., 2019). Borman and Motowidlo (1997) conceptualized employee job-related behaviors or job performances into task performance (in-role behavior) as well as contextual performance (extra-role behavior). Rotundo and Sackett (2002), in their reviews of job performance frameworks, have identified three broad dimensions, namely task performance (in-role behavior), organizational citizenship behavior (extra-role behavior), and counterproductive work behavior (deviant behavior). The new dimension, counterproductive work behavior (deviant behavior), refers to behaviors that harm the organization’s well-being. This paper conceptualizes employee behaviors into in-role, extra-role, and deviant behaviors.

The primary job requirements identified, coordinated, and controlled to achieve organizational goals are in-role behavior (Fu et al., 2015; Khoreva and Wechtler, 2018). In-role behavior is defined as “*actions specified and required by an employee’s job description and thus mandated, appraised, and rewarded by the employing organization*” (Janssen and Van Yperen, 2004, p. 369). According to Zhu (2013), extra-role behavior is employee behavior that falls beyond the formally assigned job responsibilities of individuals’ organizational positions yet is considered instrumental in promoting the organization’s operational efficiency and effectiveness. Deviant behavior has been stated as noncompliant behavior (Puffer, 1987), antisocial behavior (Robinson and O’Leary-Kelly, 1998), workplace deviance (Robinson and Greenberg, 1998), organizational misconduct (Vardi, 2001), and dysfunctional workplace behavior (Van Fleet and Griffin, 2006). Deviant behavior is defined as “*voluntary behavior that violates significant organizational norms and in doing so threatens the well-being of an organization, its members, or both*” (Robinson and Bennett, 1995, p. 556).

## Hypotheses development

### High-commitment performance management and employee behaviors

Employee voice in goal setting would make them own their job responsibilities and ensure their self-management performance (Wu et al., 2021). Conversely, Shuck et al. (2014) found that employees lose their job interest because of a lack of participation. Similarly, fair performance appraisal has a significant bearing on employee responses. Ahmed et al. (2011) discovered a statistically significant influence of performance appraisal fairness on employee citizenship behavior. In contrast, researchers noticed that improper performance appraisal is one source of employees’ counterproductive behavior in the workplace, such as absenteeism and turnover intention (Rubel and Kee, 2015b). Tziner et al. (2010) assessed that employees who experience unfair treatment related to performance appraisal could negatively impact the organization. A similar emphasis was given on the importance and positive effect of performance feedback on employee outcomes (Rosen et al., 2006). Researchers (Sommer and Kulkarni, 2012; Wu et al., 2021; Yang et al., 2021) pointed that optimistic feedback relates to employee performance advancement. However, feedback on employees’ poor performance might provoke aggressive behavior and deviant behavior (Ilgen and Davis, 2000).

Performance reward could lead to OCB (Teh et al., 2012) and task-related behavior (Shakir et al., 2014). Likewise, a poorly designed reward system might indicate deviant or unproductive behavior (Litzky et al., 2006). The relationship between HCPM and employee behaviors supports the notion of SET as well. When a company devotes in commitment-boosting HRM, employees are more inclined to respond with greater effort (McClean and Collins, 2011; Rubel et al., 2018). It is more likely that commitment-enhancing PM would be reciprocated with positive performance behaviors. Thus, we hypothesize that HCPM may affect each dimension of job behaviors.

*Hypothesis 1*: HCPM has a positive impact on in-role behavior.

*Hypothesis 2*: HCPM has a positive impact on extra-role behavior.

*Hypothesis 3*: HCPM has a negative impact on deviant behavior.

### High-commitment performance management and perceived organizational support

PM specifies a clear explanation of what, why, and how employees would work in the organization (Chowdhury, 2011). Han et al. (2010) supported that participation enhances employee psychological ownership feeling and this ownership is envisaged as organizational support for the employees. As such employee participation in performance goal setting would clearly define performance expectations set by the employee themselves and promote their ownership sense in owning work targets. DeConinck (2010) advocated that a fair perception of performance appraisal positively correlates with POS. Nasurdin et al. (2008) proved the significant positive effect of performance appraisal, one dimension of PM, on POS. Again, performance feedback was also undertaken as one key dimension of HRM having a significant positive association with POS (Tremblay et al., 2010). Allen et al. (2003) depicted that reward positively affects employee perceived support from the organization. Rhoades and Eisenberger (2002) accentuated that organizational reward is a significant antecedent of POS. Waseem (2010) analyzed the influence of reward satisfaction on employee perceptions of support from the organization.

In summary, HCPM may influence POS as employees might view commitment-focused PM as their support for performance improvement. As SET follows, such employee positive perceptions are the positive employee attitudinal exchanges for constructive formal actions presented in HCPM. Thus, the hypothesis below is developed:

*Hypothesis 4*: HCPM has a positive impact on POS.

### Perceived organizational support and employee behavior

POS is more likely to act positively in achieving organizational objectives (Rhoades and Eisenberger, 2002). According to the norm of reciprocity, employees are obligated to fulfill their responsibilities by repaying the organization in ways they find valuable (Eisenberger and Stinglhamber, 2011). Employees with enhanced POS realization perform better within and beyond their job roles (Chiang and Hsieh, 2012; Neves and Eisenberger, 2012; Casimir et al., 2014). Employees tend to reciprocate favorably to organizational support by increasing their organizational attachment (Edwards and Peccei, 2010), commitment (Chiang and Hsieh, 2012), amplifying additional role involvement (Edwards and Peccei, 2010), and decreasing actual turnover (Allen and Shanock, 2013; Rubel et al., 2020) and affective commitment (Berberoglu, 2018).

Employees can meet reciprocity expectations by lowering counterproductive/deviant behaviors in addition to elevating role-prescribed and extra-role behavior. Researchers like Rhoades and Eisenberger (2002) and Allen and Shanock (2013) had confirmed that POS negatively affect counterproductive work behavior. The relationship of POS with withdrawal behaviors such as absenteeism and voluntary turnover had been reported (Allen and Shanock, 2013; Biron, 2013). Biron (2010) revealed that a low level of support from the organization reduces employees’ feelings of obligation and pursues them to behave negatively toward the organization. Employees are more likely to exhibit in-role and extra-role behaviors and less likely to engage in deviant behaviors when POS is perceived as high. Such act of kindness like POS on the part of the employer places a duty on the part of the employees to pay it forward through both in-role and out-of-role behaviors and to lower deviant behavior. In other words, we hypothesize that the motivation for employees to conduct in-role and extra-role responsibilities that are not required or mandated by their employment as well as lower deviant behaviors is just a fair social exchange. This logic follows the hypotheses below.

*Hypothesis 5*: POS has a positive impact on in-role behavior.

*Hypothesis 6*: POS has a positive impact on extra-role behavior.

*Hypothesis 7*: POS has a negative impact on deviant behavior.

### Perceived organizational support as a mediator

Although HRM is viewed as part of organizational support, research scholars viewed HRM practices and POS as two separate constructs (Allen et al., 2003). Latorre et al. (2016) suggested that HCHRM is linked with POS because POS signals a sense of caring and well-being of employees in the form of HRM practices. There is evidence that HRM can promote more in-role and out-of-role performances when they are interpreted as showing support and procedural fairness (Tremblay et al., 2010; Rubel et al., 2020; Kee and Chung, 2021). Furthermore, prior HRM research has identified POS as a key mediating factor (Allen et al., 2003; Maan et al., 2020; Rubel et al., 2020; Hur et al., 2021). Accordingly, it is assumed that HCPM indicates organizational support for employee performance improvement, which will enhance employee in-role extra-role behaviors and reduce deviant behavior. We argue that the presence of HCPM is a source of satisfaction in itself because of the positive signals they provide. Employees are motivated to adopt positive attitudes and actions for the business when they feel supported and valued by the organization through organizational policies and practices (Allen et al., 2003). Optimistic organizational experiences (such as supportive HRM like HCHRM) will generate positive actions and attitudes to return that support, which in turn will boost perceptions of organizational support as reciprocity.

Nasurdin et al. (2008) found that POS partially mediates the relationship between HRM and employee commitment. Neves and Eisenberger (2012) confirmed that management communication only influences in-role and extra-role performance through POS. According to Cullen et al. (2014), POS significantly mediates the relationship between employees’ ability to adjust with changes and uncertainty and their satisfaction and performance. Vatankhah et al. (2017) exhibited that POS serves as the social mechanism by which HRM influences the behavioral outcomes of employees. Rubel et al. (2020) discovered a substantial mediation effect of POS between high performance HRM and employee job outcomes in the context of medical professionals in Bangladesh. However, it is unclear how POS intervenes in the interaction between HCPM and employees’ productive and counterproductive behaviors. Nevertheless, SET could be based on such conception as HCMP is a positive organizational action that could result in positive employee perceptions in the form of POS, which further generates supportive work performance of employees in exchange. Based on the previous literature supports and SET, the following hypotheses can be considered:

*Hypothesis 8*: POS mediates the effect of HCPM on in-role behavior.

*Hypothesis 9*: POS mediates the effect of HCPM on extra-role behavior.

*Hypothesis 10*: POS mediates the effect of HCPM on deviant behavior.

## Research methods

### Sample

The study included all the supervisors as population employed by registered RMG organizations (RMG) that are the members of the Bangladesh Garment Manufacturer and Exporter Association (BGMEA). However, as more than 60% of the RMG organizations were in Dhaka, the sampling frame for this study only included the supervisors of the Dhaka-based member RMG organizations. Saunders et al. (2003) and Sekaran and Bougie (2010) had commented that sampling frame is the valid representative of the total population. Dhaka was chosen as a sampling frame that would represent the RMG industry of Bangladesh. Registered RMG organizations were considered because (1) registered organizations were easy to identify and (2) the nature of the registered RMG organizations in terms of their management practices were almost similar (Bangladesh Garment Manufacturer and Exporter Association, 2019).

The study was cross-sectional, for which data were gathered in 2020. Initially, the researchers contacted 100 RMG organizations derived from the member list of the BGMEA, the authoritative body of the RMG organizations, while only 40 organizations decided to participate. Based on our initial discussion, questionnaires were delivered to the respondents through the support of the respective RMG organizations’ HR personnel. The complete work of distribution and collection of questionnaires from the responding RMG organizations took 2 months (September–October 2020).

Because of the lack of a complete list of the respondents, purposive sampling was employed to collect data from the supervisors. Purposive sampling is limited to particular groups of people who are able to supply the needed data, either because they are the only ones with it or because they meet certain criteria established by the researchers (Sekaran and Bougie, 2010). This sampling technique is used to collect data from a specific group purposively selected for the study, here were the supervisors. Based on the following three principles, the current study considered supervisors as the study respondents: (1) supervisors were controlling a group of operators who were directly involved in the fabrication activities of RMG organization; (2) supervisors who were actively working in the RMG industry for more than 5 years; and (3) supervisors who were actively working in the organization for more than 1 year.

Sekaran and Bougie (2011) recommended a sample size of 30–500 samples as adequate. Hair et al. (2019) underlined the need of using a larger sample size to avoid sampling error. The ten times rule (Barclay et al., 1995) was also used to obtain the sample size. Based on 10 times rule, the lowest number of sample size must be 10 times higher than the maximum number of arrows headed to a particular latent variable in the Partial Least square (PLS) path model. This study also used ten times rule because PLS in Structural Equation Modeling (SEM) was used in the analyses. The endogenous latent variable () was the one with the most arrowheads pointing to it and the number was 8. As a result, ten times eight, or 80, should be the sample size for this study. However, this is only a preliminary estimate of the necessary sample size (Hair et al., 2019). Moreover, in PLS, a sample size of 100 was considered suitable for data analysis and expected significant outcome. In addition to that, Hair et al. (2019) recommended that a better alternative is to carry out the model-specific power analyses for multiple regression models developed by Cohen (1992), namely G*Power, statistical power analysis. Our G* Power results revealed that 101 cases were required to identify *R*^2^ values of at least 0.25 with a 5% of error and an 80% statistical power.

Given the above-mentioned rules of thumb and the results of the power analysis, we sought 1,000 samples to ensure that this survey samples would appropriately represent or reflect the population attributes. Considering another finding based on the average external survey response of 15% (Cleave, 2020), we opted to send 1,000 questionnaires to obtain a minimum of 150 questionnaires. Thus, a total of 1,000 questionnaires were distributed equally to 40 RMGs, and 462 questionnaires were returned in total. Of 462 returned questionnaires, only 430 questionnaires were useable, giving a final response rate of 43.0%. The response rate was considered good, as Rubel and Kee (2015b) reported a response rate of 37.5% in their study on RMG in Bangladesh.

### Measures

A five-point Likert scale, with 1 denoting “Strongly Disagree” and 5 denoting “Strongly Agree,” was used to evaluate all constructs of exogenous and mediating variables. The endogenous variables, on the other hand, were evaluated using a seven-point Likert scale, with 1 denoting “Strongly Disagree” and 7 denoting “Strongly Agree.”

#### Employee behaviors

Supervisors were asked to evaluate their behaviors. Eleven items were used to assess in-role (four items with alpha 0.79), extra-role (three items with alpha 0.83), and deviant behavior (four items with alpha 0.87). Both in-role and extra-role items were adapted from Bettencourt and Brown (1997). We specifically developed four items to assess deviant behavior. The four scale items were developed based on widely referred deviant behavior content focusing on the production of deviant behavior by Demir (2011) and Colquitt et al. (2021).

#### High-commitment performance management

To measure HCPM, a total of 21 items from previously established scales were modified. Goal & participation and performance feedback dimensions were adapted from Roberts and Reed (1996) and measured by six and five items for each. Furthermore, five items measurement of performance appraisal was adapted from Korsgaard and Roberson (1995) and Lee and Jimenez (2011). Last, five items adapted from Lee and Jimenez (2011) and Daley (2017) assessed performance reward. These dimensions’ reliability scores ranged from 0.73 to 0.94. Example items were as follows: I can discuss my performance goals with my supervisor (goal and participation); My supervisor rates my performance objectively (performance appraisal); I receive timely feedback on my performance (performance feedback), and rewards are given to employees who perform (performance reward).

#### Perceived organizational support

Five items were obtained from Eisenberger et al. (2001). A shortened version was employed and found well-accepted reliable value (Kim et al., 2016; Le and Lei, 2019). The reliability of this scale was 0.89.

### Data analysis

The Smart PLS 3.2.7 version was employed to assess survey data. A sophisticated model underlined the current research, such as higher-order reflective-reflective HCPM with mediation testing. Regression analysis using SPSS was inadequate to assess such model simultaneously. Therefore, PLS-SEM was used in the current study. Researchers are now studying HRM employing PLS for data analysis and interpretation (Rubel et al., 2020, 2021). The following benefits of using PLS-SEM were emphasized by Hair et al. (2019).

- Survey data do not require always be normally distributed
- Small samples can be examined using PLS-SEM.
- PLS-SEM could be used to analyze models having both formative and reflecting constructs.
- PLS-SEM is more appropriate for evaluating mediation.

A two-step process is involved in PLS path model estimate and interpretation, and it involves evaluating the measurement model (also known as the outer model) and structural model (commonly known as the inner model) separately (Hair et al., 2019).

## Results

To get authentic results, the study addresses self-report response bias, also known as common method variance (CMV) following the proximal and methodological separation strategy suggested by Podsakoff et al. (2003). Each of the constructs in this study was measured using a different set of questionnaire instructions. The measurements of the variables were also examined using various scale formats such as five-point scale for both independent and mediating variables whereas, seven-point scale for dependent variables. In addition, to solve CMV issue, Harman’s single-factor test was used in this work. Harman’s single-factor test is well-known for measuring CMV in terms of statistical control. Using this technique, all the indicators are subjected to an exploratory factor analysis, after which the unrotated component matrix is examined to determine how many influential factors can be used to explain the variability in the variable. CMV was not a concern in the study, because the analysis of the findings showed that the first component accounted for 23.43% of the total 61.1% variation.

The participants’ demographic profile (see Table 1) showed that approximately three-quarters were male (72.5%). 80% were between 31 and 40 years old, and 71.2% had a college-level education. In terms of industry tenure, 72.6% had worked between seven to 10 years within the industry. In terms of job type, participants were mostly in the cutting section (32%), quality control section (28%), finishing section (18%), sewing section (14%), and others were from the weaving section (8%).

**Table 1:** Respondents’ demographic profile.

Demographic data	Frequency (*N* = 430)	Percentage (%)
*Age*		
21–30 years	50	11.7
31–40 years	344	80.0
40 and above	36	8.2
*Gender*		
Male	312	72.5
Female	118	27.5
*Education*		
High School (SSC)	53	12.2
College (HSC)	306	71.2
Degree	71	16.6
*Income*		
Between Tk. 20,000–Tk 25,000	227	52.72
Tk. 25,001–Tk. 30,000	133	31.0
Tk 30,001–Tk 35,000	47	11.0
Above Tk 35,000	23	5.28
*Industry experience*		
1–3 years	20	4.65
4–6 years	66	15.35
7–10 years	312	72.6
10 years and above	32	7.4
*Position (Employment Category)*		
Cutting section	137	31.86
Quality control section	121	28.14
Finishing section	77	18.0
Sewing section	60	14.0
Weaving section	34	8.0

## Hierarchical high-commitment performance management

This study implied HCPM as a second-order reflective-reflective construct made up of four first-order constructs with a total of 21 components. To confirm the reflective nature of the second-order construct, the correlations of all first-order dimensions were assessed as suggested by Hulland (1999) and Wetzels et al., (2009). Table 2 summarizes hierarchical HCPM, indicating that all the items and the constructs were associated with each other and statistically substantial at *p* < 0.01.

**Table 2:** Hierarchical HPCM.

(CR = 0.796, AVE = 0.510)			
Goal and participation	Performance appraisal	Performance feedback	Performance reward
*R*^2^ = 0.612	*R*^2^ = 0.593	*R*^2^ = 0.146	*R*^2^ = 0.687
*β* = 0.783	*β* = 0.770	*β* = 0.382	*β* = 0.829
*p* < 0.01	*p* < 0.01	*p* < 0.01	*p* < 0.01

### Measurement model

The reliability of the measures for the constructs of interest was assessed, and the reported Cronbach’s alpha coefficients were higher than 0.70. The first-order reflective constructs’ psychometric properties were evaluated in terms of convergent and discriminant validity. Three measures were deliberated to examine the convergent validity (Hair et al., 2019): (a) the factor loadings of the indicators of the individual construct, which must be statistically substantial with values greater than or equal to 0.6; (b) estimation of average variance extracted (AVE), is equal or higher than 0.5; and (c) composite reliability (CR), with values higher than or equal to 0.7. Table 3 presents a summary of the measurement model.

**Table 3:** Result of the measurement model.

Constructs	Measurement items	Loading	AVE	CR
Goal and participation (G&P)	G&P1	0.790	0.693	0.931
	G&P2	0.884		
	G&P3	0.814		
	G&P4	0.844		
	G&P5	0.845		
	G&P6	0.814		
Performance appraisal (PA)	PA1	0.736	0.576	0.871
	PA2	0.792		
	PA3	0.778		
	PA4	0.757		
	PA5	0.728		
Performance feedback (PF)	PF1	0.708	0.620	0.890
	PF2	0.854		
	PF3	0.828		
	PF4	0.755		
	PF5	0.782		
Performance reward (PR)	PR1	0.744	0.555	0.862
	PR2	0.696		
	PR3	0.716		
	PR4	0.768		
	PR5	0.798		
Perceived Orgal. Support (POS)	POS1	0.738	0.523	0.845
	POS2	0.682		
	POS3	0.711		
	POS4	0.728		
	POS5	0.754		
In-role behavior (IRB)	IRB1	0.806	0.587	0.850
	IRB2	0.682		
	IRB3	0.752		
	IRB4	0.818		
Extra-role behavior (ERB)	ERB1	0.766	0.528	0.770
	ERB2	0.718		
	ERB3	0.693		
Deviant behavior (DB)	DB1	0.728	0.707	0.906
	DB2	0.851		
	DB3	0.883		
	DB4	0.892		

Heterotrait–Monotrait (HTMT) ratio was employed to confirm the discriminant validity. Henseler et al. (2015) suggest two distinct threshold values for HTMT: 0.85 and 0.90 and recommend that the HTMT ratio delivers more accurate and significant results than the commonly used Fornell and Larcker (1981) method. We followed the minimum value of 0.85 to assess discriminant validity. Both convergent validity and discriminant validity were acceptable in this research. Table 4 demonstrates that all the values reported were within the limit.

**Table 4:** Discriminant Validity-Heterotrait–Monotrait Ration (HTMT^0.85^).

	DB	ERB	G&P	IRB	PA	PFB	POS	PWRD
DB								
ERB	0.318							
GP	0.332	0.142						
IRB	0.171	0.119	0.306					
PA	0.080	0.125	0.443	0.678				
PFB	0.161	0.134	0.526	0.615	0.711			
POS	0.263	0.384	0.223	0.575	0.556	0.372		
PWRD	0.275	0.775	0.185	0.23	0.172	0.207	0.371	
Mean	4.19	3.53	3.45	4.03	4.06	3.85	4.02	3.48
S.D.	1.44	0.84	0.85	0.67	0.57	0.68	0.59	0.80

DB, Deviant Behavior; ERB, Extra-role Behavior; GP, Goal and Participation; IRB, In-role Behavior; PA, Performance Appraisal; PFB, Performance Feedback; POS, Perceived Organizational Support, PRWD, Performance Reward.

### Structural model and hypotheses testing

To obtain a well-accepted structural model, bootstrapping of the 430 cases (the same as the original sample size) was directed with 1,000 samples. The adequacy of the structural model was evaluated by assessing the path coefficients and the effect size (*f*^2^), cross-validated redundancy (*Q*^2^), and coefficient of determination (*R*^2^; Hair et al., 2019). The anticipated model illuminated a significant proportion of the variance of endogenous latent constructs supported by the guideline of Cohen (1992), such as (0.02–0.12) weak, (0.13–0.25) moderate, and (above 0.26) substantial. In this study, the endogenous construct POS was explained by 17.2% (*Q*^2^ = 0.082) of the variance by HCPM. On the other hand, in-role behavior, extra-role behavior, and deviant behavior were explained at 0.34, 0.131, and 0.076, suggesting 34.0% (*Q*^2^ = 0.186), 13.1% (*Q*^2^ = 0.04/0.14), and 7.6% (*Q*^2^ = 0.048) of the variance, respectively, by both HCPM and POS.

First, the direct effect from HCPM to all three outcome variables, such as HCPM to in-role behavior (*β* = 0.411, *p* < 0.01), HCPM to extra-role behavior (*β* = 0.162, *p* < 0.01), and HCPMP to deviant behavior (*β* = −0.189, *p* < 0.01) was found significant. Furthermore, the relationship from HCPM to POS (*β* = 0.415, *p* < 0.01) was also found significant. Last, direct path from POS to all three outcome variables, such as in-role behavior (*β* = 0.277, *p* < 0.01), extra-role behavior (*β* = 0.190, *p* < 0.01), and deviant behavior (*β* = −0.137, *p* < 0.01) were found significant as well. The findings of the analysis exhibited that all direct hypotheses were significant (Table 5).

**Table 5:** Structural model direct effect.

Direct path	Std. Beta	Std. Error	*t*-Value	*f* ^2^	Value of *p*	Decision
HCPM → In-role behavior	0.411	0.047	8.80**	0.212	0	S
HCPM → Extra-role behavior	0.162	0.077	2.12**	0.024	0.036	S
High-Commitment PMP → Deviant behavior	−0.190	0.056	3.35**	0.032	0.001	S
High-Commitment PMP → Perceived organizational support	0.415	0.046	9.12**	0.208	0	S
Perceived organizational support → In-role behavior	0.277	0.052	5.36**	0.096	0	S
Perceived organizational support → Extra-role behavior	0.189	0.057	3.30**	0.034	0.001	S
Perceived organizational support → Deviant behavior	−0.137	0.047	2.90**	0.017	0.004	S

*p < 0.05, **p < 0.01 (based on two-tailed test with 1,000 bootstrapping).

Additionally, Preacher and Hayes’ (2008) proposal of bootstrapping indirect effect was calculated for investigating the mediation. Findings of the examination demonstrated all the mediation effects were noteworthy (Table 6), such as HCPM to POS to in-role behavior (*β* = 0.115, *p* < 0.01), HCPM to POS to extra-role behavior (*β* = 0.078, *p* < 0.01), and HCPM to POS to deviant behavior (*β* = −0.57, *p* < 0.01). As a result, each mediation hypothesis was found valid and statistically substantial.

**Table 6:** Structural model indirect effect.

Indirect path	Std. Beta	Std. Error	*t*-value	95% UL	95% LL	Value of *p*	Decision
High-Commitment PMP → POS → In-role behavior	0.115	0.026	4.424**	0.067	0.165	0.000	S
High-Commitment PMP → POS → Extra-role behavior	0.078	0.025	3.192**	0.03	0.129	0.001	S
High-Commitment PMP → POS → Deviant behavior	−0.057	0.021	2.762**	0.096	0.017	0.006	S

*p < 0.05, **p < 0.01 (based on two-tailed test with 1,000 bootstrapping).

## Conclusion and discussion

We seek to examine the relationship among the role of organizational context, HCPM, as a starting condition for workplace resources, POS, and job behaviors. We draw from SET to build our proposed model. We argue that HCPM and POS are available resources to encourage employee in-role and extra-role behaviors and minimize deviant behavior within the workplace. Employees who experience high POS from HCPM are expected to reinvest their efforts into the organization and are more likely to engage in in-role and extra-role behavior and less likely to engage in deviant behavior. We investigate the relationship between these variables in a way that few empirical investigations have been able to do. Our results support our model, whereby higher HCPM leads to POS and positive job behaviors. In this sense, we confirm that HCPM is a predicting factor of POS and job behaviors. We also confirm that POS is a significant mediator. HCPM can predict job behaviors *via* an increase in POS.

Local studies in Bangladesh have discovered a favorable correlation between HRM procedures, such as performance evaluation and employee performance behavior (Rubel and Kee, 2015b). The study results advocate that greater prominence of employee perceived HCPM stipulates improved behaviors, resulting in higher in-role and extra-role behavior and lower deviant behavior. Local research has also shown employees’ adverse behavioral outcomes such as turnover intention and turnover, indicating the inadequate application of HRM practices in which performance appraisal as a single dimension plays a significant role (Chowdhury, 2011). Therefore, it is apparent that different aspects of PM perceived by employees are significant to explore their significant behaviors in the organization. If the organization approves employees’ contribution by pertinent PM, the consequential effects might be higher productive behavior and lower deviant behavior.

**Figure 2:**
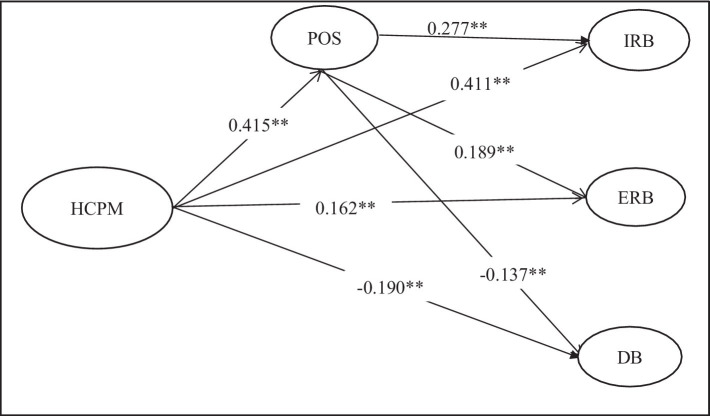
Structural model.

The study’s findings concerning employee perceived HCPM and POS demonstrate a significant positive result, in line with preceding studies where different PM practices predict POS (Tremblay et al., 2010; Gavino et al., 2012). This study’s results interpret that commitment-focused PM favored by employees has the prospective to provoke a high level of POS among employees. Employees perceived HCPM would eventually enhance their feeling of being approved by the organization as reciprocity. As such, organizations should acknowledge HCPM as an imperative predictor of employee perceived support (Figure 2).

Another finding shows a significant influence of POS on employee behavioral outcomes, including in-role behavior, extra-role behavior as productive behavior, and deviant behavior as counterproductive work behavior. Previous studies suggest that workers with a greater feeling of POS display enhanced in-role conduct (Chiang and Hsieh, 2012; Casimir et al., 2014) and extra-role behavior for both individuals and the organization (Neves and Eisenberger, 2012). Based on the concept of reciprocity, employee perceived support from the organization acts as an essential predictor of expected behaviors from the employees (Eisenberger and Stinglhamber, 2011). Therefore, from the findings of the present study and the previous literature supports, it can be proclaimed that employees’ perceived support from the organization affects various performance outcomes within the direction of social exchange.

Moreover, the study finding portrays that POS also mediates the relationship between HCPM and employee behavioral outcomes (extra-role, in-role, and deviant). This finding supports the notion of POS’s significance for the employees in the organization (Allen and Shanock, 2013; Tremblay and Landreville, 2015). The study results convey that employees perceived HCPM could enhance their extra-role and in-role behavior and minimize deviant behavior by enhancing employee-required supports from the organization. Thus, the organization should emphasize HCPM as commitment enhancers and POS builders to ensure social approval of employee contribution. In doing so, the organization should practice HCPM and POS to increase both extra-role and in-role behavior and reduce deviant behavior to enhance the development of both individuals and organizations.

Our findings suggest that HCPM should be promoted to influence employees’ perceptions of organizational support and job behaviors. Those experiencing high levels of POS have also been shown to have positive job behaviors. To conclude, this study has contributed to a new extent in solving the debates around the role of individual HRM practice in influencing employee outcomes. It is done by developing and testing a mediation model on the relationship between HCPM and employee productive behavior and deviant behavior. This study adds POS specifically as a significant attitudinal component and link in the causal chain. The association between perceived HCPM and two different employee outcomes, namely performance behavior and deviant act, is revealed to be mediated by POS. The results are in line with the core tenets of SET, which contend that firms that can foster a culture of reciprocity will probably influence their workforces to behave positively. Our study unequivocally demonstrates that employees take into account their perceptions of HCPM to improve their comprehension of POS, performance, and motivation to act in accordance with organizational expectations. Therefore, the role of HCPM is expected to contribute to the development of a comprehensive PM approach that would not only assess employee performances but also develop the sense of POS in promoting positive employee behaviors.

## Theoretical implications

This research augments the literature on how specific HRM practices influence employee behavioral outcomes theoretically. The findings of this study have threefold implications. HCPM stimulates POS and enhances both extra-role and in-role behavior, and minimizes employees’ deviant behavior in the workplace. Therefore, HCPM makes it easier to establish the position of the single dimension of HCHRM practices in HRM literature, defending the idea that critically analyzing individual HRM practices is important (Haines et al., 2010). Second, this study represents the social approval notion of SET as well (Homans, 1961). Execution of HCPM recognizes and approves employee contribution and elicits their positive behaviors to the organization. In another way, less emphasis on PM indicates a lack of social approval of employee contribution that may further instigate employees’ negative behavior to the organization.

Third, this research’s distinct finding explicates some of the “black box” workings: HCPM enhances employee extra-role and in-role behavior and reduces deviant behavior through POS. In our consideration of the “black box” clarifying how HRM practices impact employee behavioral outcomes in the organization, we have seen that HCPM stimulates related behavioral outcomes of the employees by promoting the feeling of employee perceived support from the organization. Overall, the explorations of HCPM with its different dimensions would suggest a guideline to the future researchers in assembling the specific dimensions of other individual HRM or HCHRM practices and the mechanism through which individual HRM or HCHRM practices relate to employee outcomes.

## Practical implications

Our empirical context affords us the unique opportunity to make several contributions. First, our study reiterates that RMG managers should be prepared to use HCPM in the labor-oriented manufacturing sector for sustained performances. RMG managers need to be aware that the implementation of HCPM may lead to positive supervisor performances. Second, our study suggests that RMG organizations should prioritize POS as it may help to enhance the effect of HCPM on employee positive performances (in-role and extra-role) and reduce deviant behavior. Third, RMG organizations may also consider targeting supervisors who hold a longstanding relationship with operating employees, as supervisors have a spillover effect on them under their supervision. Their positive performances will influence and motivate operating employees’ performances. The RMG organizations should be aware of such potential positive spillover effects experienced by the operating employees from their supervisors. The impact of HCPM on supervisory performance, both directly and indirectly, is somewhat demonstrated in our research. HCPM, performance-focused practices, promote positive supervisor performances and minimize their deviant behavior through POS.

Since Bangladeshi RMG organizations are labor-intensive and labor productivity is the main indicator of organizational productivity, supervisors play the critical roles in making their subordinates high performers. So, it will be significant for the management of the organization to manage the behaviors of supervisors first who can ensure the goal-oriented behaviors of their subordinates. In this regard, organization should focus on implementing structured PM practices to ensure enhanced productivity and declined deviant behaviors of the supervisors who would eventually ensure such practices for getting expected outcomes from their subordinates. Additionally, policymakers might establish rules and incentive programs to promote appropriate PM practices because doing so may help to improve POS among employees. Understanding the POS factors in the forms of HCPM dimensions with goal setting and participation, performance appraisal, feedback on the performance, and appropriate rewards against performance, government should streamline proper PM guidelines for the labor-intensive industries to deal with sustained productive performances. Last, our outcomes support the growth of both local and international position of our RMG industry to achieve higher rank through making their employees more productive and task oriented in the organization.

## Limitations and future research directions

The present research has numerous suggestions for the academics and researchers to conduct future research, some of which connect to this study’s limitations. In this research, only RMG organizations in Dhaka city were taken into account. Thus, the generalizability of this research finding was somewhat limited. Other restraints of this study reported about the respondents. For example, data were gathered only from the supervisor level. The study results would be more consistent if future researchers collect data considering employees working across the industry to reconcile their observations on HCPM and its outcomes. From this research, an organization might obtain information about how different dimensions of HCPM enhance a supervisor’s performance and reduce deviant behavior through increased POS from their self-report measurement. Future research can investigate how other individual HCHRM practices shape employees’ attitudes and behavior. The study showed the education and experience of the supervisors under the demographic profile and their impact were not assessed in the respondent’s responses as well as in the model. Therefore, this could be considered another limitation of the current research. Future researchers might take this issue into consideration and employ these two attributes as predictors of employee work outcomes in the organization and test their effects. In future, researchers could use PLS and AMOS to calculate and show all the criteria including maximum shared variance (MSV) and average shared variance (ASV) for discriminant validity.

Future studies can look at the potential contributions of other individual dimensions of HCHRM in an elaborative perspective to examine their impact on the employee attitudes and behavior in various organizational contexts. Furthermore, based on the specific characteristics of HRM practices revealed in the prior literature, it might be advised for future researchers to look at the higher-order paradigm of those specific dimensions. We suggest that future researchers and practitioners explore the higher-order constructs of particular HRM practices with their respective dimensions and their applicability in the organizations because making higher-order allows for more theoretical parsimony and reduces the complexity of the model.

## Data availability statement

The original contributions presented in the study are included in the article/supplementary material, further inquiries can be directed to the corresponding author.

## Ethics statement

Ethical review and approval was not required for the study on human participants in accordance with the local legislation and institutional requirements. Written informed consent from the patients/participants OR patients/participants legal guardian/next of kin was not required to participate in this study in accordance with the national legislation and the institutional requirements.

## Author contributions

MR and NR was involved in all the steps and procedures followed in this study, conceptualization, reviewing the literature, finalizing research methodology, data collection and analysis, and writing and reviewing the original draft. DK provided technical assistance, actively participated in all the steps followed in this study, and helped in conceptualization and improving this draft. YD played an important role in reviewing literature and hypotheses development. All authors contributed to the article and approved the submitted version.

## Conflict of interest

The authors declare that the research was conducted in the absence of any commercial or financial relationships that could be construed as a potential conflict of interest.

## Publisher’s note

All claims expressed in this article are solely those of the authors and do not necessarily represent those of their affiliated organizations, or those of the publisher, the editors and the reviewers. Any product that may be evaluated in this article, or claim that may be made by its manufacturer, is not guaranteed or endorsed by the publisher.

## Figures and Tables

**Table:** 

Constructs	
**Goal and Participation**	**Perceived Organizational Support**
I can discuss my performance goals with my supervisor	My organization shows great concern for me
I can talk to my supervisor about the goals that I want to pursue in my career	My organization extends help to perform my job in the best way
I can openly discuss work-related problems with my supervisor	Help is available when I have a problem
I can have an influence on how my performance goals were designed	My organization takes pride in my success at work
My supervisor expects more of me when we set goals together	My organization cares about my opinion
My supervisor and I jointly develop my performance goals	**In-role Behavior**
**Performance Appraisal**	I perform all those tasks that are required of me
My supervisor rates my performance objectively	I fulfill responsibilities as specified in the job description
If I perform well, it is reflected in my performance appraisal	I meet formal performance requirements
The performance appraisal process in my organization is free from bias	I adequately complete all of the expected tasks
The performance appraisal in my organization is based on factual information	**Extra-role Behavior**
The performance appraisal in my organization is verifiable	I voluntarily assist my colleagues, even if it means going beyond job requirements
**Performance Feedback**	I often go above and beyond the call of duty when serving in the organization
I receive timely feedback on my performance	I run the extra mile to help my colleagues
I receive an adequate amount of feedback on my performance	**Deviant Behavior**
My supervisor tells me what I do wrong	I come to work late or leave early
My supervisor provides clear feedback on my performance	I work slow and withhold efforts at work
My supervisor provides frequent feedback on my performance	I take excessive breaks during working hours
**Performance Reward**	I have trouble showing up for meetings or work
Rewards are given to the employees who perform	
Employees are appropriately rewarded based on performance	
I understand how my pay relates to my performance	
Performance is used to determine a potential salary increase	
Performance is used to determine a potential promotion opportunity	
